# Supplementary material for: Synaptic mechanisms for associative learning in the cerebellar nuclei
Source: Nat Commun. 2023 Nov 20;14:7459. doi: 10.1038/s41467-023-43227-w (PMC10662440; doi:10.1038/s41467-023-43227-w)
Supplement: Supplementary file 1 — Supplementary Information [file 41467_2023_43227_MOESM1_ESM.pdf]

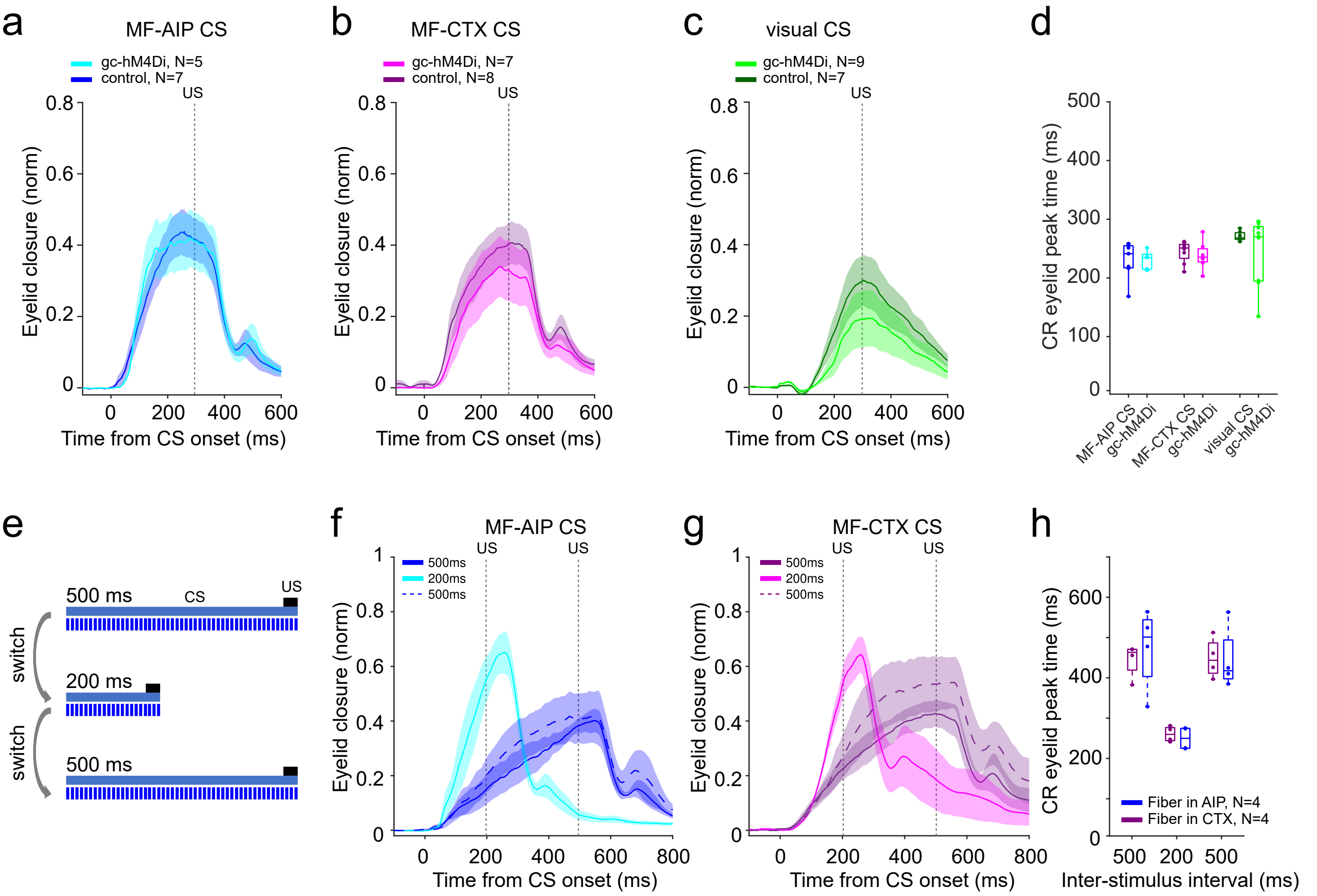

Supplementary Fig. 1. Timing of conditioned responses during granule cell inhibition and shifting of inter-stimulus intervals. a-c Average eyelid traces from granule cell-expressing DREADD mice (lighter colors) and respective non-DREADD-expressing controls (darker colors), injected with CNO prior to training sessions. Averages include CS-only trials from the last session of training. Vertical gray line indicates the expected time of US delivery. **a** Animals trained to MF-AIP CS. **b** Animals trained to MF-CTX CS. **c** Animals trained to a visual CS. **d** CR eyelid peak times comparing DREADD mice versus non-DREADD-expressing controls, conditioned with different types of CS. **e** Schematic of experimental design: mice were trained to shifting ISIs of 500, 200 and back to 500 ms. The two stimuli co-terminated for all experiments, as is typical for delay conditioning. **f-g** Average eyelid traces from mice trained to a MF-AIP CS or a MF-CTX CS. Traces include CS-only trials from sessions with a ISI of 500 ms (blue line), 200 ms (cyan line) and back to 500 ms (dashed blue line). **h** Average CR peak time for MF-AIP CS and MF-CTX CS animals trained to shifting ISIs: 500 ms, 200 ms and 500 ms. No differences were found across ISIs between the two groups (respectively: P = 0.62, P = 0.56, P = 0.95, unpaired t-test, two-tailed). In d and h, dots represent individual animals. Boxes indicate median and 25th-75th percentiles, and whiskers extend to the most extreme data points. In all other graphs data are shown as mean ± SEM. Source data are provided as a Source Data file.

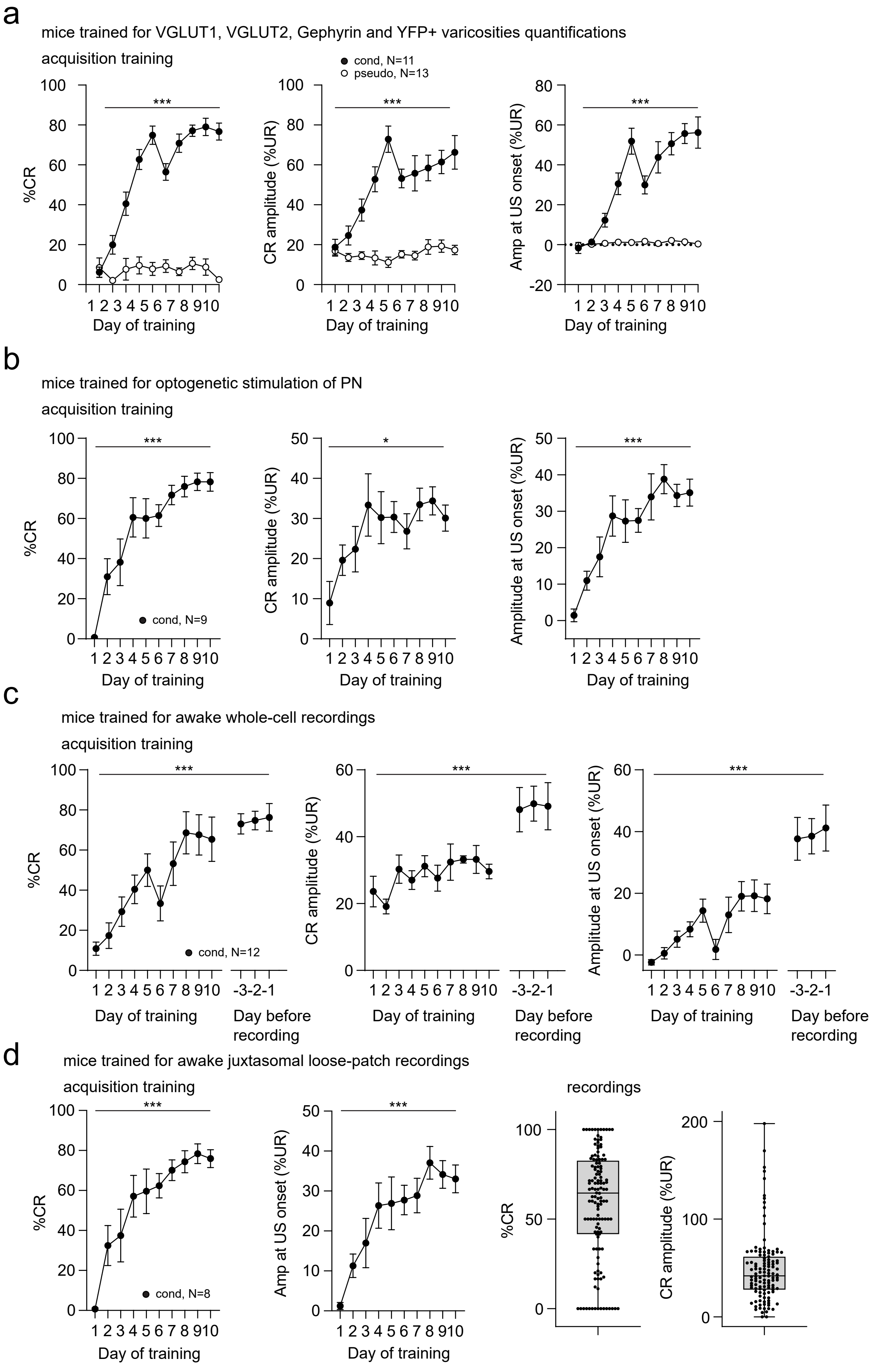
a
mice trained for VGLUT1, VGLUT2, Gephyrin and YFP+ varicosities quantifications
acquisition training
cond, N=11
pseudo, N=13
%CR
CR amplitude (%UR)
Amp at US onset (%UR)
Day of training
b
mice trained for optogenetic stimulation of PN
acquisition training
cond, N=9
%CR
CR amplitude (%UR)
Amplitude at US onset (%UR)
Day of training
c
mice trained for awake whole-cell recordings
acquisition training
cond, N=12
%CR
CR amplitude (%UR)
Amplitude at US onset (%UR)
Day of training
Day before recording
d
mice trained for awake juxtasomal loose-patch recordings
acquisition training
recordings
cond, N=8
%CR
Amp at US onset (%UR)
CR amplitude (%UR)
Day of training

Supplementary Fig. 2. Learning curves of conditioned and pseudo-conditioned mice. a Learning curves of conditioned (cond) and pseudo-conditioned (pseudo) mice for histological quantifications of biomarkers during acquisition training. Conditioned, but not pseudo-conditioned mice showed increases in %CR (main-effect of day: $F_{(9,23.878)}$ = 51.525, P < 0.001, main-effect of group: $F_{(1,24.066)}$ = 383.255, P < 0.001, interaction day x group: $F_{(9,23.878)}$ = 48.090, *** P < 0.001, linear mixed models), CR amplitude (main-effect of day: $F_{(9,23.325)}$ = 15.710, P < 0.001, main-effect of group: $F_{(1,23.408)}$ = 79.379, P < 0.001, interaction day x group: $F_{(9,23.325)}$ = 18.365, *** P < 0.001, linear mixed models) and amplitude at US onset (main-effect of day: $F_{(9,23.992)}$ = 158.821, P < 0.001, main-effect of group: $F_{(1,24.006)}$ = 127.739, P < 0.001, interaction day x group: $F_{(9,23.992)}$ = 153.423, *** P < 0.001, linear mixed models). **b** Learning curves of conditioned mice for optogenetic stimulation of the pontine nuclei (PN), showing increasing %CR ($F_{(3.098,24.79)}$ = 16.27, *** P < 0.001, one-way repeated-measures ANOVA), CR amplitude ($F_{(2.885,23.08)}$ = 4.385, * P = 0.015, one-way repeated-measures ANOVA) and amplitude at US onset ($F_{(4.139,33.11)}$ = 10.78, *** P < 0.001, one-way repeated-measures ANOVA). **c** Learning curves of conditioned mice for awake whole-cell recordings, showing increasing %CR ($F_{(12,87.223)}$ = 6.889, *** P < 0.001, linear mixed models), CR amplitude ($F_{(12,72.079)}$ = 3.6, *** P < 0.001, linear mixed models) and amplitude at US onset ($F_{(12,84.439)}$ = 6.069, *** P < 0.001, linear mixed models). After acquisition training, mice were placed in the electrophysiology setup and trained for >3 days to maintain high levels of performance (day before recording). **d** Learning curves of conditioned mice for awake juxtasomal recordings, showing increasing %CR ($F_{(9,49.188)}$ = 7.134, *** P < 0.001, linear mixed models) and amplitude at US onset ($F_{(9,47.846)}$ = 4.599, *** P < 0.001, linear mixed models; left panels). Average performance across all recordings (n = 132) from the same mice (N = 8) during juxtasomal recordings (right panels), CRs occurred in 57.95 ± 2.67% of trials across all recordings and had amplitudes of 48.38 ± 3.1 %UR. Boxplots show median and 25th-75th percentiles, whiskers show minimum and maximum values. Data in all other graphs is shown as mean ± SEM. Source data are provided as a Source Data file.

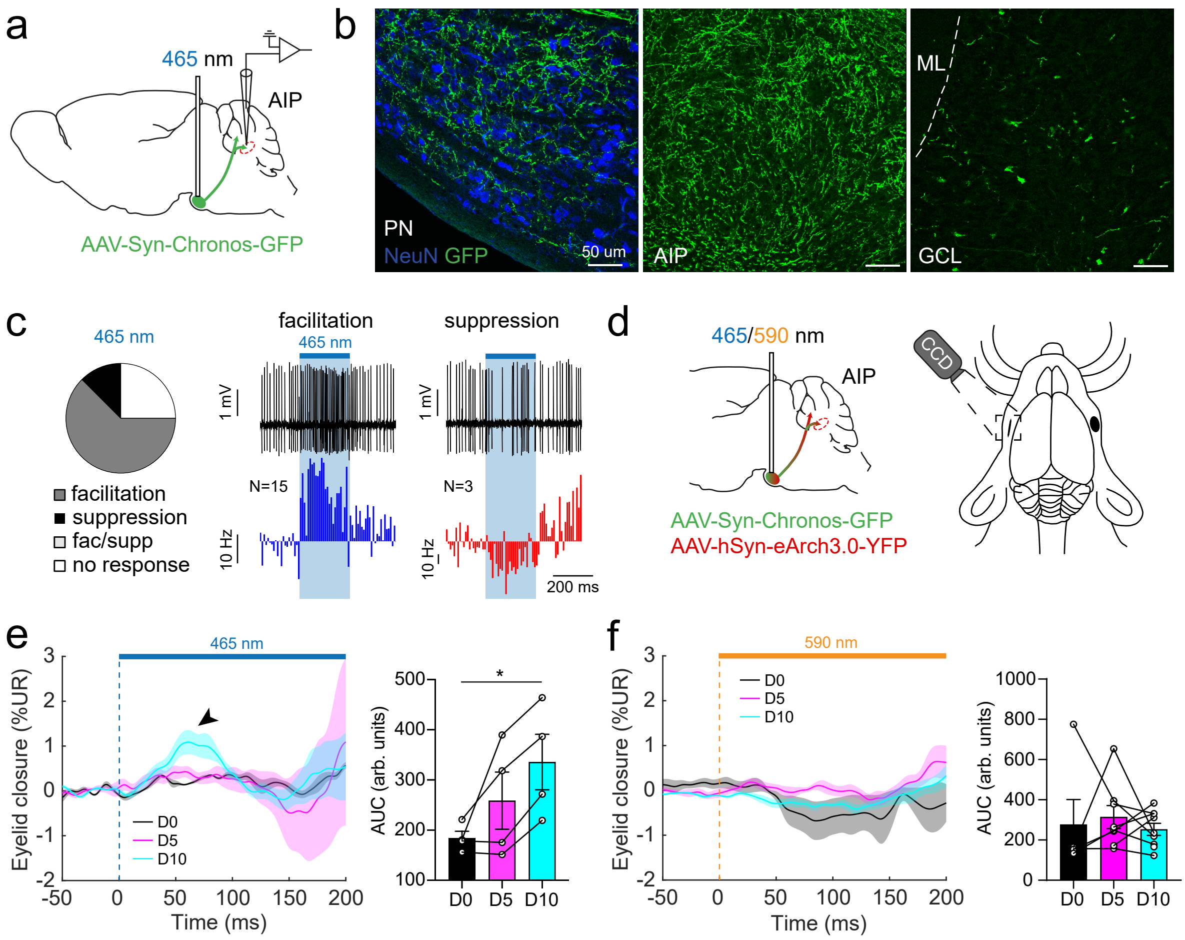

Supplementary Fig. 3. Optogenetic stimulation of pontine nuclei neurons leads mainly to spike facilitation and eyelid movements after conditioning. a Schematic of optogenetic stimulation of pontine nuclei (PN) neurons giving rise to MFs targeting the cerebellum. AAV-Chronos-GFP was injected bilaterally in the PN and optic fiber implants were placed directly above. **b** Expression of GFP+ axons in the PN, anterior interposed nucleus (AIP) and granule cell layer (GCL). No GFP expression in the molecular layer (ML) indicating that no climbing fibers were labeled. **c** Juxtasomal recordings (n = 24) showing 62% spike facilitation, 13% spike suppression and 25% no response neurons. Example spike traces show representative spike facilitation and suppression neuron, with the blue bar and shaded area indicating 465 nm light illumination. Histograms show average spike rates across spike facilitation and suppression neurons. Short-latency spike changes occur directly after PN activation. **d** Schematic of dual optogenetic stimulation. AAV-Chronos-GFP and AAV-eArch3.0-YFP were co-injected bilaterally in the PN followed by optic fiber implants. Eyelid movements were recorded while optogenetically stimulating PN neurons. **e** Average eyelid movements of mice (N = 4) during optogenetic stimulation of PN neurons with 465 nm (blue) light on day (D) 0, D5 and D10 of conditioning. Area under the curve (AUC) of short-latency eyelid movements (0-150 ms after light onset) showing a significant increase in magnitude of eyelid closure on D10 (arrowhead; AUC at D0 vs. D5 vs. D10: 184.2 ± 13.57 vs. 258.9 ± 57.1 vs. 335.7 ± 55.31, $F_{(2,6)}$ = 6.883, * P = 0.028, D0 vs. D10: P = 0.023, one-way repeated-measures ANOVA with Tukey's multiple comparisons test). **f** Average eyelid movements

of mice (N = 9) during optogenetic stimulation of PN neurons with 590 nm (amber) light on D0, D5 and D10 of conditioning. AUC of eyelid movements (0-150 ms after light onset) does not show significant changes over the course of conditioning ($F_{(1.594, 8.764)}$ = 0.2812, P = 0.714, linear mixed models). Data are shown as mean ± SEM. Source data are provided as a Source Data file.

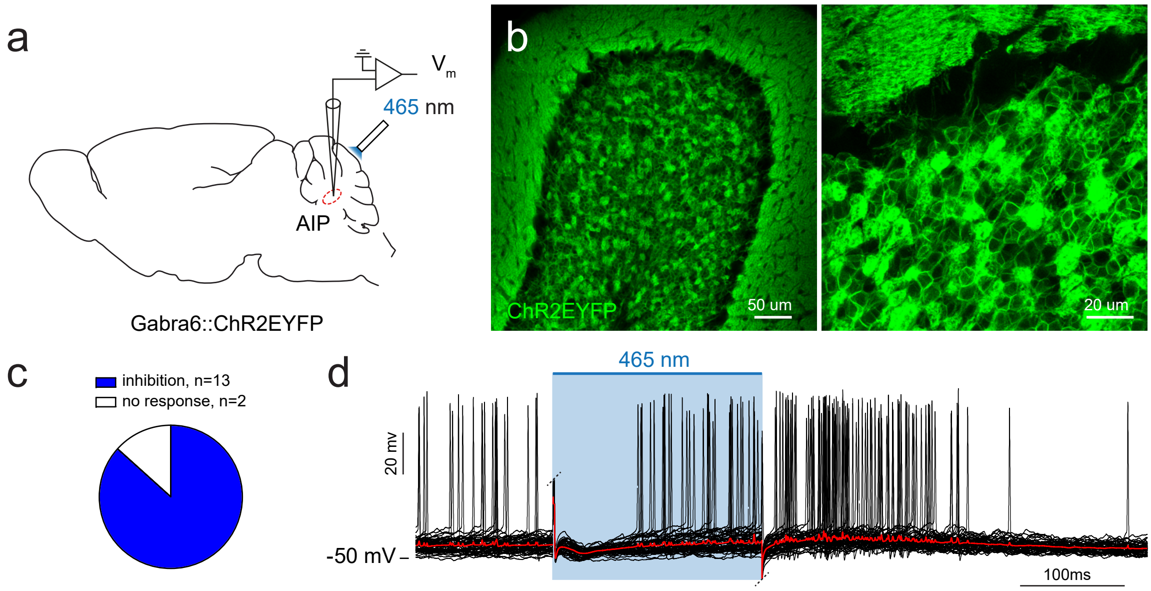

Supplementary Fig. 4. Cerebellar nuclei neurons show mainly inhibition during optogenetic activation of granule cells. a Schematic of whole-cell recordings of anterior interposed nucleus (AIP) neurons during optogenetic stimulation of granule cells with 465 nm (blue) light in Gabra6::ChR2EYFP transgenic mice. **b** Expression of ChR2EYFP in granule cells and parallel fibers in the molecular layer. ChR2EYFP expression was verified in one mouse. **c** AIP neurons show predominantly mean decreases in spiking activity (86.7%) immediately after onset of optogenetic light. **d** Example representative neuron showing inhibitory responses to granule cell activation. Raw traces (black) with average trace (red) superimposed. The blue bar and shaded area indicate 465 nm light illumination. Electrical noise artifacts at light onset and offset are clipped. Source data are provided as a Source Data file.

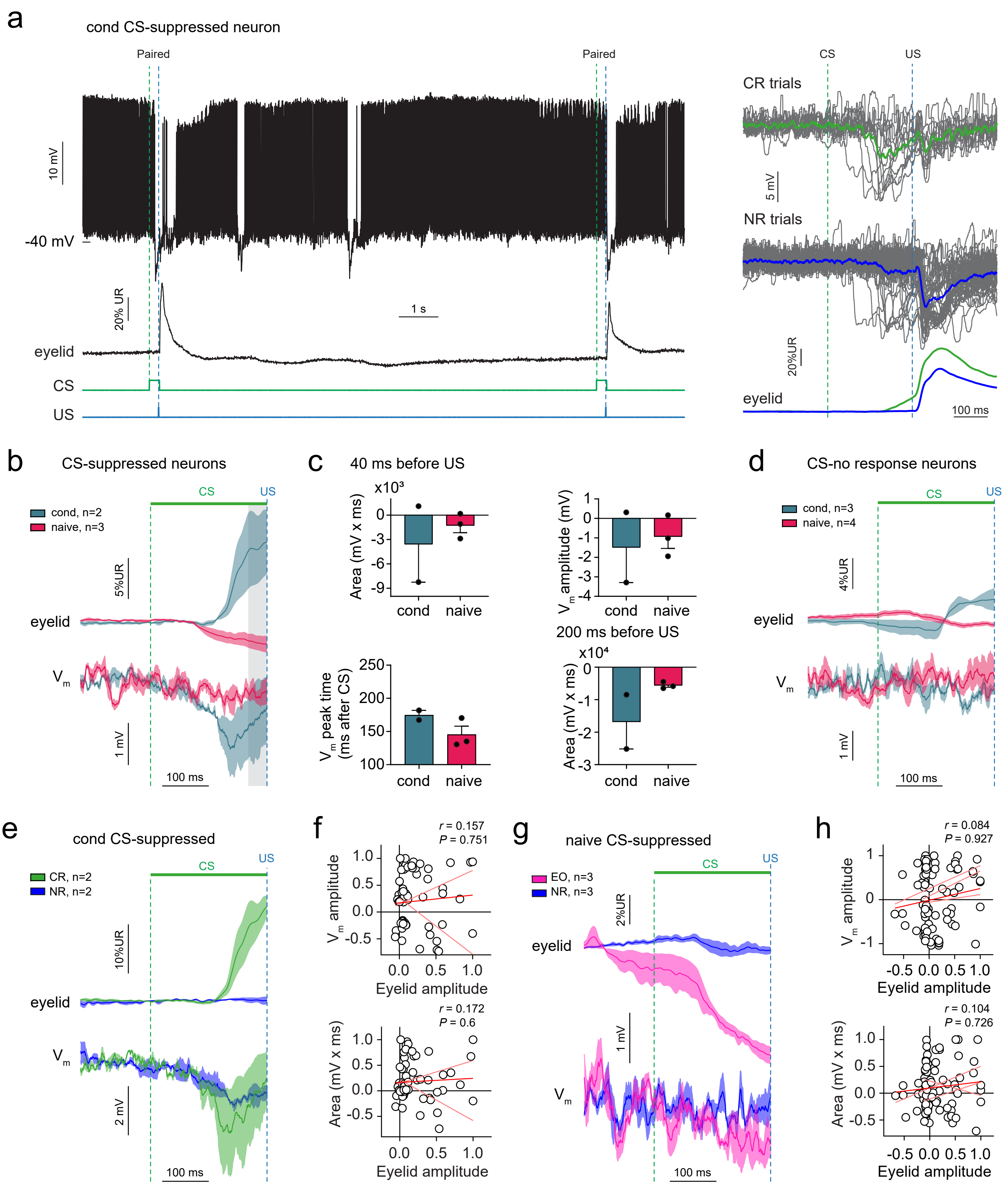
a
cond CS-suppressed neuron
Paired
Paired
10 mV
-40 mV
20% UR
1 s
eyelid
CS
US
CS
US
CR trials
5 mV
NR trials
20%UR
eyelid
100 ms
b
CS-suppressed neurons
cond, n=2
naive, n=3
CS
US
5%UR
eyelid
Vm
1 mV
100 ms
c
40 ms before US
x10³
Area (mV x ms)
cond
naive
Vm amplitude (mV)
200 ms before US
x10⁴
Vm peak time (ms after CS)
d
CS-no response neurons
cond, n=3
naive, n=4
4%UR
e
cond CS-suppressed
CR, n=2
NR, n=2
10%UR
2 mV
f
r = 0.157
P = 0.751
Eyelid amplitude
r = 0.172
P = 0.6
g
naive CS-suppressed
EO, n=3
NR, n=3
2%UR
h
r = 0.084
P = 0.927
r = 0.104
P = 0.726

Supplementary Fig. 5. Membrane potential responses in CS-suppressed cerebellar nuclei neurons. **a** Example trace of a representative conditioned CS-suppressed neuron. V_m hyperpolarizations with temporary cessation of spike activity occur during CS-US paired trials and between trials. Larger average hyperpolarizations appear on conditioned response (CR) trials compared to no response (NR) trials during the CS-US interval (right panels). CS: conditioned stimulus, US: unconditioned stimulus. **b** Eyelid and V_m response averages for CS-suppressed neurons, separating conditioned and naive neurons. Gray area indicates the last 40 ms before the US used calculation of area in (c). **c** Graphs showing quantifications of V_m responses shown in (b). No statistical comparison due to the small sample size. **d** Eyelid and V_m response averages for CS-no response neurons, separating conditioned and naive neurons. These neurons do not show CS-evoked V_m responses, irrespective of eyelid movements. **e** Average eyelid and V_m traces on CR and NR trials of CS-suppressed neurons in conditioned mice. **f** Normalized V_m amplitude (top panel) and normalized area during the last 50 ms of the CS-US interval (bottom panel) are not correlated with normalized eyelid amplitude across pooled trial data from conditioned CS-suppressed neurons (V_m amplitude: r = 0.157, P = 0.751; area: r = 0.172, P = 0.6, Spearman, two-tailed, Bonferroni-corrected, n = 72 trials, N = 2 neurons). **g** Average eyelid and V_m traces on eyelid opening (EO) and NR trials of CS-suppressed neurons in naive mice. **h** Normalized V_m amplitude (top panel) and normalized area during the last 50 ms of the CS-US interval (bottom panel) are not correlated with normalized eyelid amplitude across pooled trial data from naive CS-suppressed neurons (V_m amplitude: r = 0.084, P = 0.927; area: r = 0.104, P = 0.726, Spearman, two-tailed, Bonferroni-corrected, n = 78 trials, N = 3 neurons). Linear regression lines for each individual neuron (pink) and for pooled trials of all neurons (red) in f and h. Graphs and traces show mean ± SEM. Source data are provided as a Source Data file.

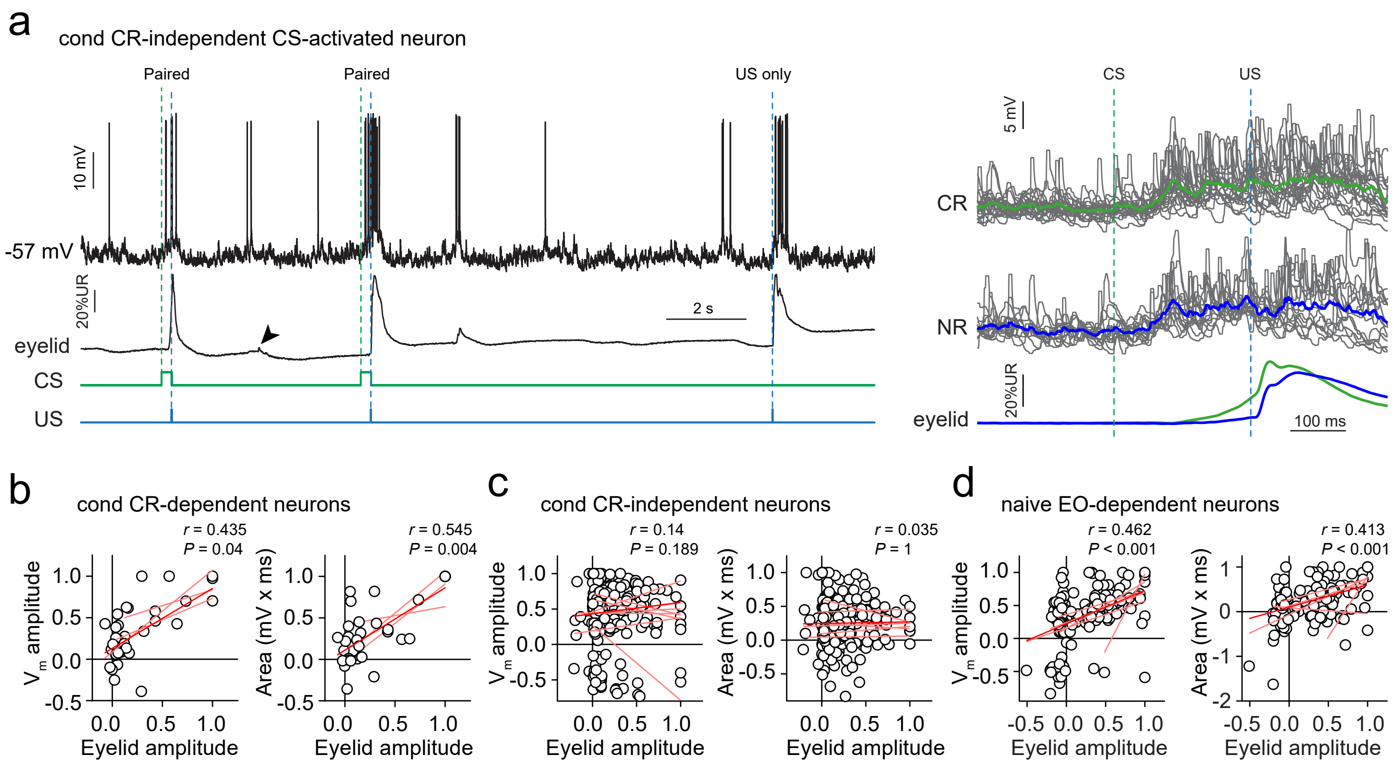

Supplementary Fig. 6. Membrane potential responses in CS-activated CR-dependent and CR-independent cerebellar nuclei neurons. a Example trace of a representative conditioned CS-activated CR-independent neuron. V_m depolarizations and spike activity occurs in response to conditioned (CS) and unconditioned stimuli (US), in addition to spontaneous eyelid movements (arrowhead). V_m depolarizations are similar on conditioned response (CR) and no response (NR) trials, and are therefore not dependent on eyelid movement (CR-independent; right panel). An example of a CR-dependent neuron is shown in Fig. 3d. **b** Normalized V_m amplitude (left panel) and normalized area during the last 50 ms before the US are both significantly correlated with the normalized eyelid amplitude across pooled trial data from conditioned CR-dependent CS-activated neurons (V_m amplitude: $r = 0.435$, $P = 0.04$; area: $r = 0.545$, $P = 0.004$, Spearman, two-tailed, Bonferroni-corrected, n = 34 trials, N = 3 neurons). **c** Normalized V_m amplitude (left panel) and normalized area during the last 50 ms before the US are not correlated with the normalized eyelid amplitude across pooled trial data from conditioned CR-independent CS-activated neurons (V_m amplitude: $r = 0.14$, $P = 0.189$; area: $r = 0.035$, $P = 1$, Spearman, two-tailed, Bonferroni-corrected, n = 203 trials, N = 6 neurons). **d** Normalized V_m amplitude (left panel) and normalized area during the last 50 ms before the US are both significantly correlated with the normalized eyelid amplitude across pooled trial data from naive EO-dependent CS-activated neurons (V_m amplitude: $r = 0.462$, $P < 0.001$; area: $r = 0.413$, $P < 0.001$, Spearman, two-tailed, Bonferroni-corrected, n = 135 trials, N = 8 neurons). In panels b-d, linear regression lines for each individual neuron are indicated in pink and for pooled trials of all neurons in red. Source data are provided as a Source Data file.

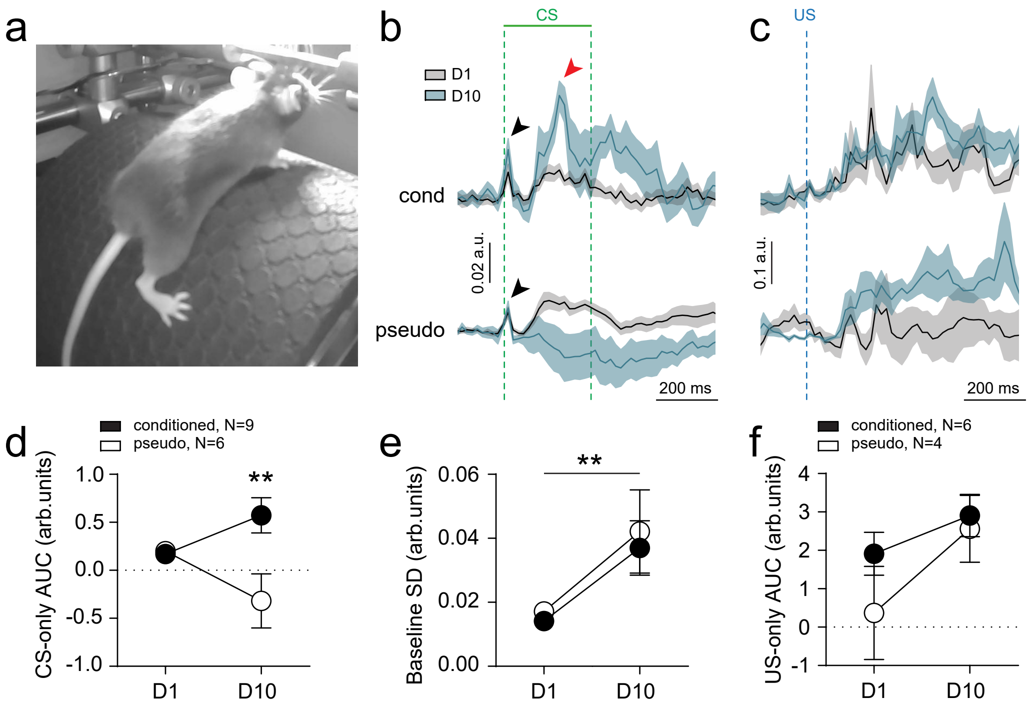

Supplementary Fig. 7. Mice develop whole-body movements during conditioning. a Video recordings of infrared-illuminated mice during conditioning or pseudo-conditioning on a treadmill. **b** Whole-body movements visualized as movement index for conditioned (N = 9) and pseudo (N = 6) mice. Both groups show short-latency movements to conditioned stimulus (CS) onset, both on day 1 (D1) and day 10 (D10; black arrowhead). Conditioned, but not pseudo mice, develop movements during the period of the CS-US interval where conditioned eyelid movements occur on D10 (red arrowhead). **c** Whole-body movements during the unconditioned stimulus (US) does not change during conditioning or pseudo-conditioning. **d** Areas under the curve (AUC) on CS-only trials, comparing D1 versus D10 between groups, show that significant body movements develop on D10 in conditioned mice (main-effect of training day: $F_{(1,13)} = 0.098$, $P = 0.76$, main-effect of group: $F_{(1,13)} = 7.296$, $P = 0.018$, interaction-effect training day vs. group : $F_{(1,13)} = 6.355$, $P = 0.026$, D10 cond vs. pseudo: ** $P = 0.002$, 2-way repeated measure ANOVA with Šídák's multiple comparisons test, cond: N = 9 mice, pseudo: N = 6 mice). **e** Standard deviation (SD) of baseline body movements, occurring before CS or US stimuli, shows a significant increase for both groups on D10 (main-effect of training day: $F_{(1,13)} = 9.857$, ** $P = 0.008$, main-effect of group: $F_{(1,13)} = 0.302$, $P = 0.592$, interaction-effect training day vs. group: $F_{(1,13)} = 0.019$, $P = 0.891$, 2-way repeated measure ANOVA). **f** Body movements of conditioned (N = 6) and pseudo (N = 4) mice in response to the US do not significantly change (main-effect of training day: $F_{(1,8)} = 3.41$, $P = 0.102$, main-effect of group: $F_{(1,8)} = 2.016$, $P = 0.193$, interaction-effect training day vs. group: $F_{(1,8)} = 0.481$, $P = 0.508$, 2-way repeated measure ANOVA). Graphs and traces show mean ± SEM. Source data are provided as a Source Data file.

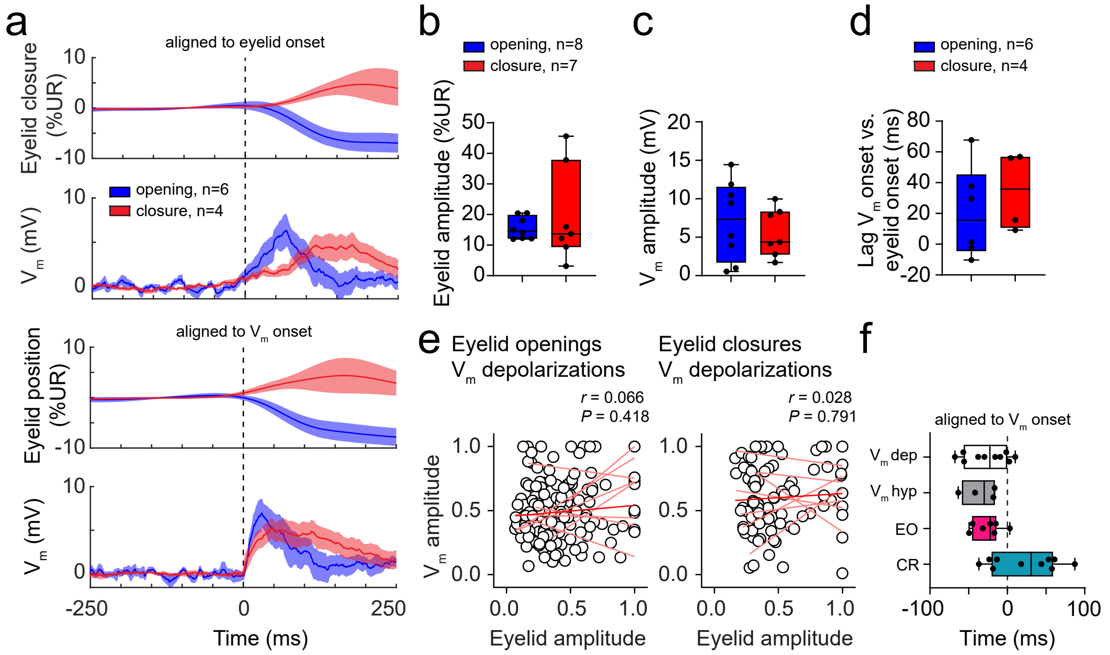

Supplementary Fig. 8. Membrane potential dynamics during spontaneous eyelid movements. a Average eyelid and V_m traces of neurons showing V_m depolarizing responses during spontaneous eyelid openings and eyelid closures. Traces are aligned to the onset of the eyelid movement (top panels) or aligned to the V_m onset (bottom panels). Only neurons in which the onsets of V_m responses and eyelid movements could be determined are included. **b-c** Graphs showing quantifications of the absolute eyelid amplitude (b) and V_m amplitude (c), which show no difference in amplitude of the eyelid movement ($t_{(13)}$ = 0.728, *P* = 0.479, unpaired *t*-test, two-tailed, n = 8 opening/7 closure neurons) and V_m response ($t_{(13)}$ = 0.674, *P* = 0.512, unpaired *t*-test, two-tailed). **d** Average lag of V_m response relative to the onset of eyelid opening or closure is comparable ($t_{(8)}$ = 0.757, *P* = 0.471, unpaired *t*-test, two-tailed, n = 6 opening/4 closure neurons). **e** Normalized V_m amplitude and normalized eyelid amplitude show no correlation for eyelid openings (*r* = 0.066, *P* = 0.418, Spearman, two-tailed, n = 155 openings in 8 neurons) or eyelid closures (*r* = 0.028, *P* = 0.791, Spearman, two-tailed, n = 91 closures in 7 neurons). Linear regression lines for each individual neuron are indicated in pink and for pooled trials of all neurons in red. **f** Onset of eyelid movements relative to V_m onset. Lags were comparable between neurons showing V_m depolarizations and hyperpolarizations ($t_{(12)}$ = 0.563, *P* = 0.584, unpaired *t*-test, two-tailed, n = 10 depolarization/4 hyperpolarization neurons). Lags were also comparable for spontaneous eyelid movements and naive EOs ($t_{(19)}$ = 0.313, *P* = 0.758, unpaired *t*-test, two-tailed, n = 14 spontaneous/7 EO neurons). Spontaneous eyelid movements as well as CS-evoked eyelid openings (EO) start before onset of the V_m response. CRs start on average after the V_m response. Boxplots show median and 25th-75th percentiles, whiskers show minimum and maximum values. All other panels show mean ± SEM. Source data are provided as a Source Data file.

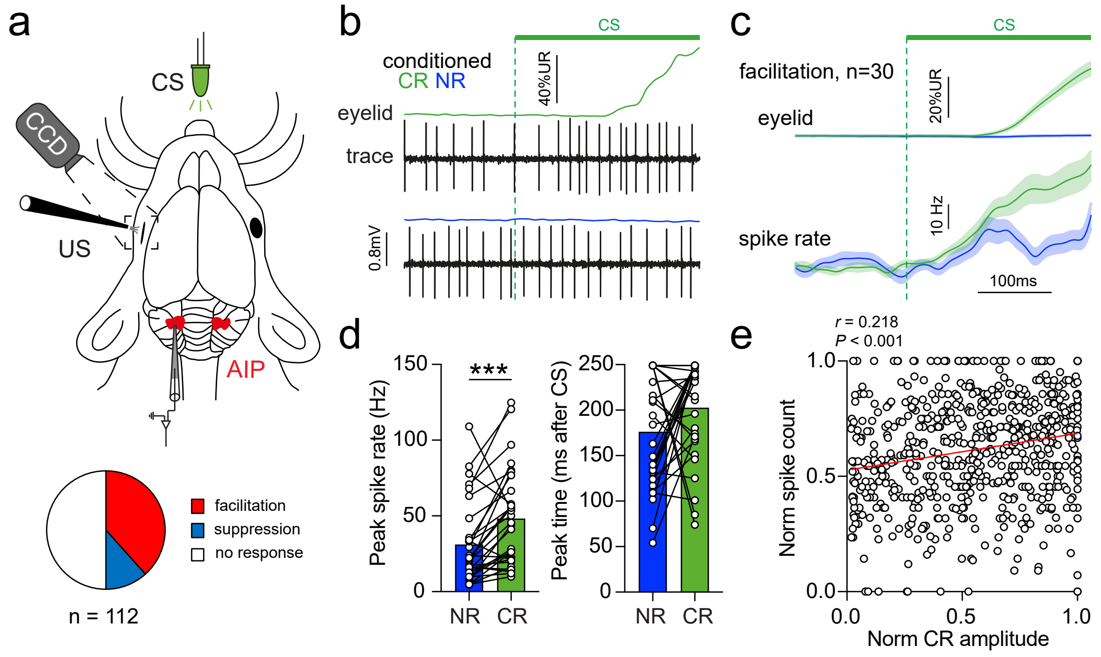

Supplementary Fig. 9. Spike activity during conditioned response and no response trials in conditioned mice. a Schematic of *in vivo* juxtasomal recordings from AIP neurons in head-fixed mice on a treadmill during EBC (top). Distribution of spike responses to the conditioned stimulus (CS; bottom) shows that the predominant response is spike facilitation. **b** Example of a representative spike facilitation neuron showing increased spike activity during a conditioned response (CR) trial, but no change in spike activity during a no response (NR) trial. **c** Average of all spike facilitation neurons showing eyelid movements (top) on CR and NR trials with normalized spike rates (bottom). **d** Average peak spike rates on NR and CR trials. Significantly higher spike rates are observed during CR trials (Z = -341, *** P < 0.001, Wilcoxon, two-tailed, n = 30 neurons), but the time of peak spike rate after CS onset is comparable (Z = -137, P = 0.083, Wilcoxon, two-tailed). **e** Pooled correlation analysis of all CR trials shows a significant correlation between number of spikes during the CS-US interval and CR amplitude (r = 0.218, P < 0.001, Spearman, two-tailed, n = 648 trials, N = 30 neurons). Data are shown as mean ± SEM. Source data are provided as a Source Data file.

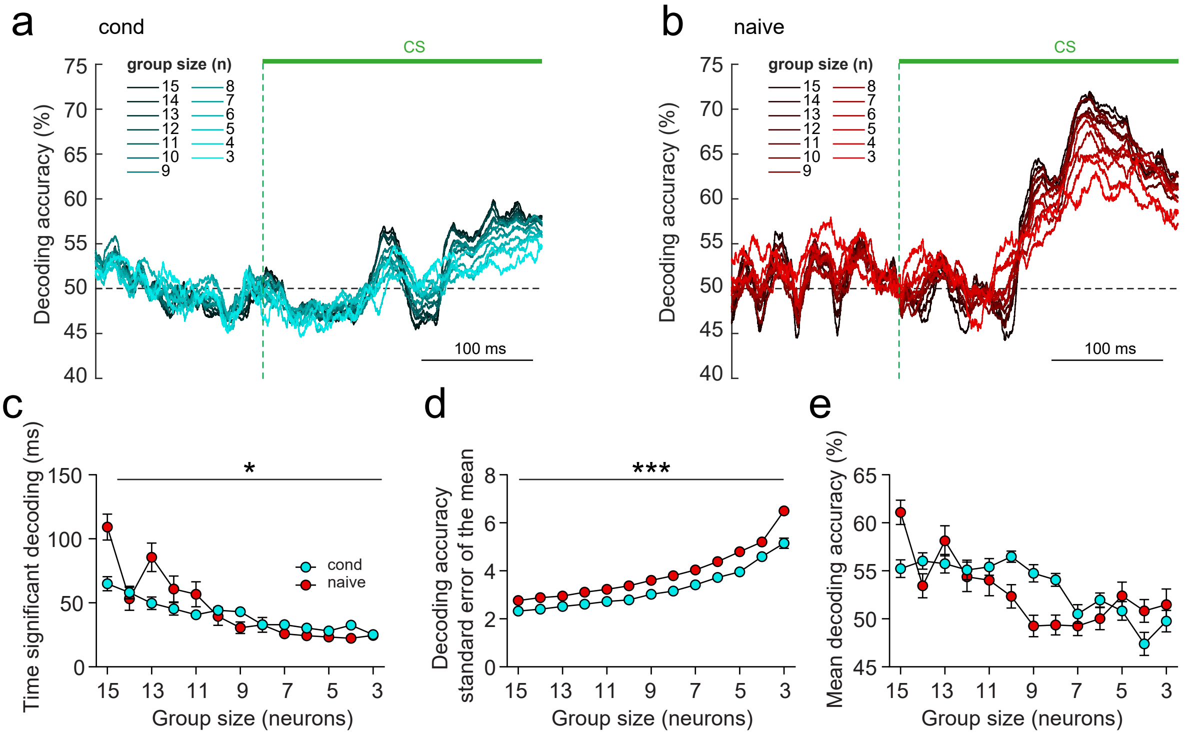

Supplementary Fig. 10. Decoding of eyelid movements based on V_m data decreases with decreasing group sizes. a Average decoding accuracy (29 iterations; CR versus NR trials) over the course of a trial based on V_m data, for different group sizes of conditioned neurons (n = 15 to n = 3 neurons). **b** Average decoding accuracy (29 iterations; EO versus NR trials) over the course of a trial based on V_m data, for different group sizes of naive neurons (n = 15 to n = 3 neurons). **c** Total time that above-average decoding was achieved decreases with decreasing group sizes and plateaus to ~25 ms for both groups ($F_{(1,348)}$ = 4.016, * P = 0.046, 2-way repeated measures ANOVA). **d** Variability in decoding accuracy, shown as standard error of the mean (SEM) across iterations, increases with decreasing group sizes ($F_{(1,348)}$ = 237.2, *** P < 0.001, 2-way repeated measures ANOVA). **e** Mean decoding accuracy during the time period of significant above-chance decoding does not significantly decrease with decreasing group sizes ($F_{(1,348)}$ = 1.435, P = 0.232, 2-way repeated measures ANOVA). Data are shown as mean ± SEM. Source data are provided as a Source Data file.

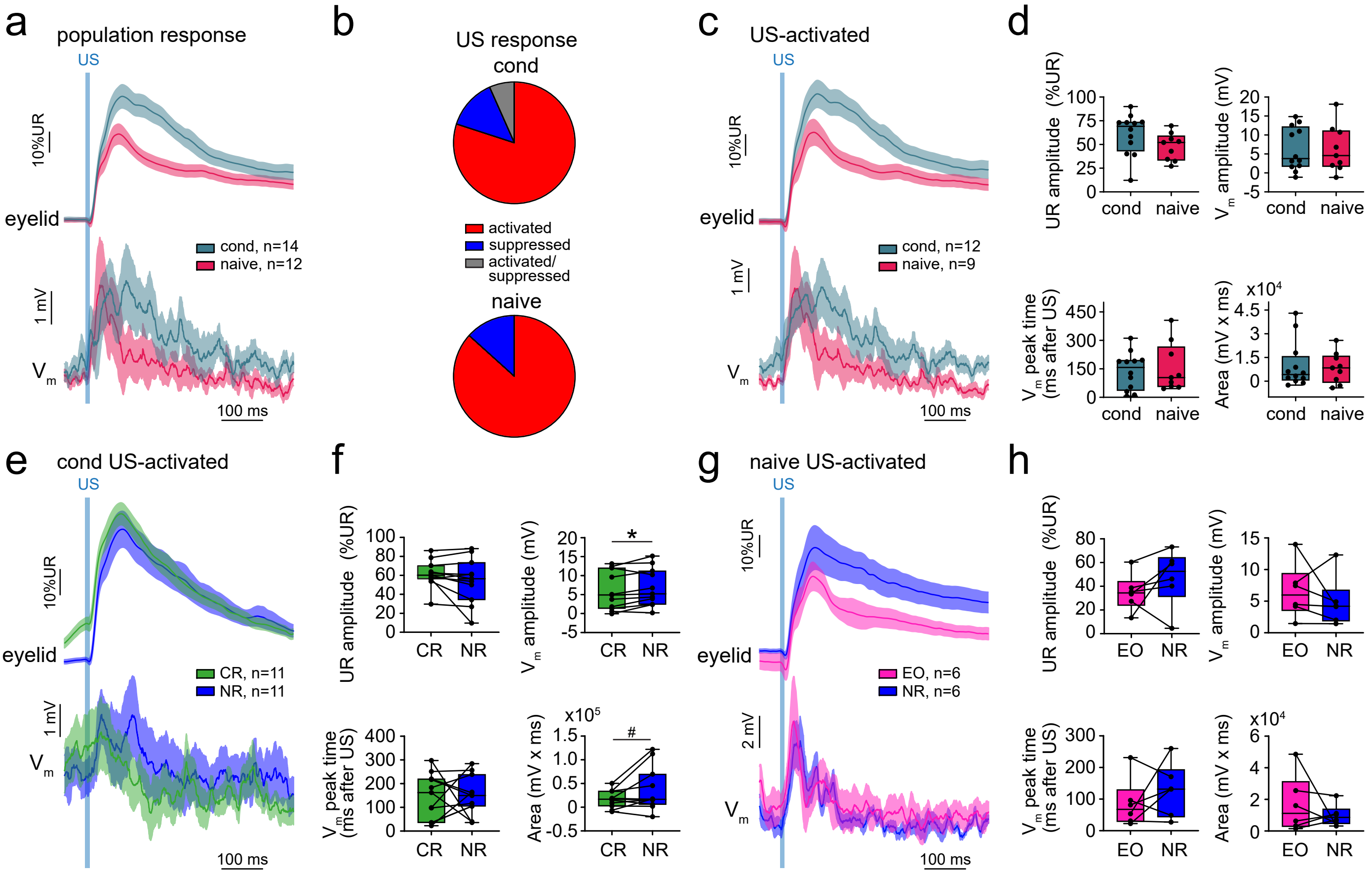

Supplementary Fig. 11. Membrane potential dynamics and spike coding during the unconditioned stimulus. **a** Population eyelid and V_m response averages across all neurons, showing predominantly V_m depolarizations in conditioned and naive neurons coinciding with the unconditioned eyelid closure response (UR). **b** Distribution of US-evoked V_m responses. For both groups the predominant response classification is US-activation. **c** Eyelid and V_m response averages of US-activated neurons. **d** Graphs showing quantifications of US responses shown in (c). There are no differences in UR amplitude, V_m amplitude, peak time and area following the US between conditioned and naive neurons. **e** Eyelid and V_m response averages on paired trials of US-activated conditioned neurons, separating CR and NR trials. **f** Graphs showing quantifications of US responses shown in (e). A preceding CR significantly lowers the amplitude of the US-evoked V_m depolarization (amplitude: $t_{(10)}$ = 2.368, * P = 0.039, paired t-test, two-tailed, area: $t_{(10)}$ = 2.057, # P = 0.067, paired t-test, two-tailed, n = 11 neurons). **g** Eyelid and V_m response averages on paired trials of US-activated naive neurons, separating EO and NR trials. **h** Graphs showing quantifications of US responses shown in (g). There are no differences in V_m amplitude and area following the US between EO and NR trials (amplitude: $t_{(5)}$ = 0.914, P = 0.403, paired t-test, two-tailed, area: $t_{(5)}$ = 0.863, P = 0.428, paired t-test, two-tailed, n = 6 neurons), indicating that a preceding EO does not influence the US-evoked V_m response. Boxplots show median and 25th-75th percentiles, whiskers show minimum and maximum values. All other panels show mean ± SEM. Source data are provided as a Source Data file.

Supplementary Table 1. Electrophysiological properties of neurons recorded in awake mice.

Group	ID	Mouse ID	Side	Depth (µm from pia)	V_m rest (mV)	Spike amp (mV)	Spike HW (ms)	Rs (MΩ)	Ri (MΩ)	Rm (MΩ)	Cm (pF)
Cond	1	1	Ipsi	n/a	-59.0	29.5	0.3	43.0	111.4	68.3	75.8
	2	2	Ipsi	1910	-60.4	27.9	1.8	24.7	89.5	64.9	35.9
	3	3	Ipsi	1538	-69.8	36.8	1.0	43.7	518.1	474.4	3.5
	4	4	Ipsi	1885	-68.7	23.9	0.8	73.6	621.1	547.5	7.7
	5	5	Ipsi	1741	-64.8	35.3	0.5	36.4	211.0	174.6	161.0
	6	6	Contra	1771	-68.7	22.0	1.0	37.1	980.4	943.3	18.8
	7	6	Contra	1830	-71.1	n/a	n/a	52.1	135.7	83.5	1.4
	8	7	Contra	1956	-61.3	21.0	0.9	57.5	305.8	248.3	62.6
	9	7	Contra	1694	-70.2	38.9	2.1	39.6	840.3	800.8	2.7
	10	8	Contra	1757	-58.4	18.2	0.5	61.2	308.6	247.4	87.1
	11	9	Contra	1701	-69.8	22.2	1.1	42.6	425.5	382.9	5.0
	12	10	Ipsi	1900	-73.2	n/a	n/a	57.3	110.9	53.5	3.8
	13	10	Ipsi	2005	-72.1	n/a	n/a	71.2	119.0	47.8	13.1
	14	11	Ipsi	n/a	-74.7	n/a	n/a	61.8	89.4	27.6	147.5
	15	12	Ipsi	1608	-68.8	n/a	n/a	50.0	125.8	75.8	6.6
Mean ± SEM				1792 ± 38.3	-67.4 ± 1.4	27.6 ± 2.3	1.0 ± 0.2	50.1 ± 3.5	332.8 ± 74.4	282.7 ± 75.1	42.2 ± 13.8
Naive	1	1	Ipsi	2244	-68.1	24.2	0.5	63.1	115.6	52.6	73.9
	2	1	Contra	1600	-70.0	15.2	1.6	74.6	568.2	493.6	5.4
	3	2	Ipsi	1978	-66.7	27.5	0.9	29.1	213.2	184.1	21.6
	4	3	Ipsi	1757	-67.4	26.3	1.0	28.3	190.8	162.5	38.2
	5	4	Contra	n/a	-55.3	32.1	0.3	31.5	57.9	26.4	163.4
	6	5	Ipsi	1712	-65.9	27.7	1.0	37.7	216.9	179.2	44.2
	7	6	Ipsi	1526	-69.2	27.7	1.6	35.4	352.1	316.7	4.6
	8	6	ipsi	1637	-68.9	n/a	n/a	61.8	136.4	74.6	23.8
	9	7	Ipsi	1968	-67.3	14.9	2.2	62.1	317.5	255.3	32.6
	10	8	Contra	2081	-68.9	51.8	1.3	30.2	885.0	854.8	17.0
	11	8	Contra	1903	-68.9	48.9	1.0	20.6	1754.4	1733.8	23.3
	12	9	Contra	1795	-67.3	25.3	0.9	51.8	125.3	73.5	51.7
	13	9	Contra	1775	-74.8	n/a	n/a	49.1	78.4	29.2	4.4
	14	10	Ipsi	n/a	-70.7	40.0	1.1	41.0	452.5	411.5	5.5
	15	11	Ipsi	1850	-73.8	n/a	n/a	53.8	97.5	43.7	90.9
Mean ± SEM				1813 ± 52.9	-68.2 ± 1.1	30.1 ± 3.3	1.1 ± 0.1	44.7 ± 4.1	370.8 ± 114.4	326.1 ± 116.1	40.0 ± 11.0
