# Supplementary material for: Synaptic mechanisms for associative learning in the cerebellar nuclei
Source: Nat Commun. 2023 Nov 20;14:7459. doi: 10.1038/s41467-023-43227-w (PMC10662440; doi:10.1038/s41467-023-43227-w)
Supplement: Supplementary file 3 — Description of Additional Supplementary Files [file 41467_2023_43227_MOESM3_ESM.pdf]

Description of Additional Supplementary Files

Supplementary Movie 1. Example recording of a CS- and US-activated conditioned neuron. Awake whole-cell recording (real-time speed) during eyeblink conditioning, showing simultaneous camera recording of the eye, quantification of eyelid opening and CS and US stimulus signals, followed by a single paired trial (10x slower).

Supplementary Movie 2. Example recording of a CS-suppressed and US-activated & suppressed naive neuron. Awake whole-cell recording (real-time speed) during eyeblink conditioning, showing simultaneous camera recording of the eye, quantification of eyelid opening and CS and US stimulus signals, followed by a single paired trial (10x slower).

Supplementary Movie 3. Whole-body movements develop only in conditioned mice. Camera recordings of an example conditioned and pseudo-conditioned mouse, comparing responses to the conditioned stimulus (CS) on training day 1 vs. day 10. Only conditioned mice develop whole-body movements after CS onset.
