# Supplementary material for: Synaptic mechanisms for associative learning in the cerebellar nuclei
Source: Nat Commun. 2023 Nov 20;14:7459. doi: 10.1038/s41467-023-43227-w (PMC10662440; doi:10.1038/s41467-023-43227-w)
Supplement: Supplementary file 7 — Reporting Summary [file 41467_2023_43227_MOESM7_ESM.pdf]

nature portfolio

Corresponding author(s): Chris I. De Zeeuw

Last updated by author(s): 02-11-2023

Reporting Summary

Nature Portfolio wishes to improve the reproducibility of the work that we publish. This form provides structure for consistency and transparency in reporting. For further information on Nature Portfolio policies, see our Editorial Policies and the Editorial Policy Checklist.

Statistics

For all statistical analyses, confirm that the following items are present in the figure legend, table legend, main text, or Methods section.

n/a	Confirmed	
☐	☒	The exact sample size (*n*) for each experimental group/condition, given as a discrete number and unit of measurement
☐	☒	A statement on whether measurements were taken from distinct samples or whether the same sample was measured repeatedly
☐	☒	The statistical test(s) used AND whether they are one- or two-sided<br>*Only common tests should be described solely by name; describe more complex techniques in the Methods section.*
☐	☒	A description of all covariates tested
☐	☒	A description of any assumptions or corrections, such as tests of normality and adjustment for multiple comparisons
☐	☒	A full description of the statistical parameters including central tendency (e.g. means) or other basic estimates (e.g. regression coefficient) AND variation (e.g. standard deviation) or associated estimates of uncertainty (e.g. confidence intervals)
☐	☒	For null hypothesis testing, the test statistic (e.g. *F*, *t*, *r*) with confidence intervals, effect sizes, degrees of freedom and *P* value noted<br>*Give P values as exact values whenever suitable.*
☒	☐	For Bayesian analysis, information on the choice of priors and Markov chain Monte Carlo settings
☒	☐	For hierarchical and complex designs, identification of the appropriate level for tests and full reporting of outcomes
☐	☒	Estimates of effect sizes (e.g. Cohen's *d*, Pearson's *r*), indicating how they were calculated

Our web collection on statistics for biologists contains articles on many of the points above.

Software and code

Policy information about availability of computer code

Data collection	Eyeblink conditioning data was collected using custom-written code in LabView (National Instruments) and stored locally or written to a database in Microsoft Access. Electrophysiology data was collected using pCLAMP and Multiclamp Commander (Molecular Devices). Confocal imaging data was acquired using LAS AF software of the SP5 (Leica).
Data analysis	Eyeblink conditioning data (MDMT) was analyzed using the commercially-available BLINK software (Neurasmus). Eyeblink conditioning data (camera) was analyzed using custom-written code in MATLAB R2011b (Mathworks). In vivo electrophysiology data was analyzed using custom-written code in MATLAB R2011b and R2022b (Mathworks) and Clampfit 10 (Molecular Devices). ImageJ was used for analysis of confocal images. Statistics were performed in MATLAB R2011b and R2022b (Mathworks), SPSS v22 (IBM) and Prim 5 and 9 (Graphpad). General analyses were done in Microsoft Excel. The code used for the analysis of our experiments are available at https://github.com/BroRobin/SynMechCN.

For manuscripts utilizing custom algorithms or software that are central to the research but not yet described in published literature, software must be made available to editors and reviewers. We strongly encourage code deposition in a community repository (e.g. GitHub). See the Nature Portfolio guidelines for submitting code & software for further information.

Data

Policy information about availability of data

All manuscripts must include a data availability statement. This statement should provide the following information, where applicable:

- Accession codes, unique identifiers, or web links for publicly available datasets
- A description of any restrictions on data availability
- For clinical datasets or third party data, please ensure that the statement adheres to our policy

Source data are provided with this paper. Given the complexity of data sets and large file sizes, the data files are available from the corresponding authors upon request.

Human research participants

Policy information about studies involving human research participants and Sex and Gender in Research.

Reporting on sex and gender	Not applicable.
Population characteristics	Not applicable.
Recruitment	Not applicable.
Ethics oversight	Not applicable.

Note that full information on the approval of the study protocol must also be provided in the manuscript.

Field-specific reporting

Please select the one below that is the best fit for your research. If you are not sure, read the appropriate sections before making your selection.

[x] Life sciences [ ] Behavioural & social sciences [ ] Ecological, evolutionary & environmental sciences

For a reference copy of the document with all sections, see nature.com/documents/nr-reporting-summary-flat.pdf

Life sciences study design

All studies must disclose on these points even when the disclosure is negative.

Sample size	No prior calculation of sample sizes was performed, since the success rate and the effect sizes of parameters calculated from vivo whole-cell recordings was unknown. Sample sizes were determined to be sufficient based on previous studies using a similar technique (e.g., https://doi.org/10.7554/eLife.07290 or https://doi.org/10.1016/j.neuron.2015.08.039). Sample sizes for extracellular, optogenetic and histological experiments were based on sample sizes in similar published studies (e.g., https://doi.org/10.7554/eLife.28132; https://doi.org/10.1523/JNEUROSCI.0511-13.2013). All our datasets (extracellular, whole-cell, optogenetic) were in good agreement with each other and with results acquired in other studies (e.g., https://doi.org/10.7554/eLife.28132; https://doi.org/10.1038/s41593-018-0129-x), confirming adequate sample sizes.
Data exclusions	During the eyeblink data analysis, eyelid traces were excluded if they had unstable baselines (>5x SD baseline) or an unstable unconditioned response amplitude (>0.5 x CV). The whole session was excluded if more than 75% of trials was invalid. Whole cell recordings were discarded if Ra exceeded 100 MΩ or if spike amplitudes were lower than 10 mV. Neurons were identified based on occurrence of clear spikes (spontaneous or current-induced). Only good traces with a non-drifting Vm were selected for further analysis.
Replication	No measures were taken to verify the reproducibility of the findings due to the level of difficulty of the experiments.
Randomization	Mice were randomly allocated to experimental groups. Mice for quantification of structural differences were age and sex-balanced within batches.
Blinding	Blinding was performed at two stages of the analysis. For the electrophysiology data analysis, onsets of conditioned responses/eyelid openings and Vm responses were performed blind to trial condition. For the VGLUT1, VGLUT2 and Gephyrin data analysis, as well as all other structural quantifications, quantifications were performed by a blind experimenter to the experimental group. Condition of the mice (trained/pseudo) was announced after completion of the analysis. Investigators were not blind to the experimental group during behavioral trainings, since it was not possible to start the acquisition software without knowing which training protocol needed to be performed.

Reporting for specific materials, systems and methods

We require information from authors about some types of materials, experimental systems and methods used in many studies. Here, indicate whether each material, system or method listed is relevant to your study. If you are not sure if a list item applies to your research, read the appropriate section before selecting a response.

Materials & experimental systems

n/a	Involved in the study
☐	☒ Antibodies
☒	☐ Eukaryotic cell lines
☒	☐ Palaeontology and archaeology
☐	☒ Animals and other organisms
☒	☐ Clinical data
☒	☐ Dual use research of concern

Methods

n/a	Involved in the study
☒	☐ ChIP-seq
☒	☐ Flow cytometry
☒	☐ MRI-based neuroimaging

Antibodies

Antibodies used

Primary Antibodies:
Rabbit anti-HA, 1:400, Cell Signaling, cat. # 3724
Mouse anti-calbindin, 1:1500, Swant, cat. # 300
Mouse anti-gephyrin, 1:500, Synaptic Systems, cat. # 147021
Rabbit anti-GFP, 1:1000, Chemicon, cat. # AB3080
Chicken anti-GFP, 1:1000, Aves labs, cat. # GFP-1020
Mouse anti-NeuN, 1:500, Merck Millipore, cat. # MAB377
Rabbit anti-VGluT1, 1:1000, Synaptic Systems, cat. # 135302
Guinea pig anti-VGluT1, 1:500, Synaptic Systems, cat. # 135304
Guinea pig anti-VGluT2, 1:1000, Synaptic Systems, cat. # 135404

Secondary Antibodies and Steptavidins:
Donkey anti-mouse Cy3, 1:1000, Jackson Immunoresearch, cat. # 715-165-150
Goat anti-chicken Alexa Fluor 488, 1:1000, ThermoFisher Scientific, cat. # A-11039
Donkey anti-guinea pig Cy3, 1:1000, Jackson Immunoresearch, cat. # 706-165-148
Donkey anti-rabbit Alexa Fluor 647, 1:1000, ThermoFisher Scientific, cat. # A-31573
Donkey anti-rabbit Alex Fluor 488, 1:1000, ThermoFisher Scientific, cat. # A-32790
Streptavidin Alex Fluor 488, 1:1000, ThermoFisher Scientific, cat. # S11223
Streptavidin Cy3, 1:1000, Jackson Immunoresearch, cat. # 016-160-084

Various:
DAPI Mounting Medium (abcam), cat. # ab104139

Validation

Rabbit anti-HA (Cell Signaling, cat. # 3724) has been validated by the manufacturer by SimpleChIP® Enzymatic Chromatin IP Kits, as well as by previous studies showing specific immunofluorescence staining on mouse tissue, as reported in the manifacturer's website.
Mouse anti-calbindin (Swant, cat. # 300) immunolabels a subpopulation of neurons in the normal brain with high efficiency but does not stain in the brain of calbindin D-28k knock out mice (validation statement on the manufacturer's website).
Mouse anti-gephyrin (Synaptic Systems, cat. # 147021) has been validated in gephyrin knock-out mice (manufacturer's statement).
Rabbit anti-GFP (Chemicon, cat. # AB3080) has been validated for use in ELISA, immunocytochemistry, immunohistochemistry, western blot for the detection of Green Fluorescent Protein (manufacturer's statement).
Chicken anti-GFP (Aves labs, cat. # GFP-1020) has been validated by western blot analysis and immunohistochemistry using transgenic mice expressing the GFP gene product (manufacturer's statement).
Mouse anti-NeuN (Merck Millipore, cat. # MAB377) has been published and validated for use in Flow Cytometry, immunocytochemistry, immunofluorescence, immunohistochemistry, immunohistochemistry (Paraffin), immunoprecipitation, Western Blotting (manufacturer's statement).
Rabbit anti-VGluT1 (Synaptic Systems, cat. # 135302) has been validated in VGluT1 knock-out mice (manufacturer's statement).
Guinea pig anti-VGluT1 (Synaptic Systems, cat. # 135304) has been validated in VGluT1 knock-out mice (manufacturer's statement).
Guinea pig anti-VGluT2 (Synaptic Systems, cat. # 135404) has been validated in VGluT2 knock-out mice (manufacturer's statement).

Animals and other research organisms

Policy information about studies involving animals; ARRIVE guidelines recommended for reporting animal research, and Sex and Gender in Research

Laboratory animals

Adult C57Bl/6J mice (4-14 weeks old, institutional colony and from Janvier Laboratories) were used in this study. Mice were socially housed where possible, at room temperature (20-22 degrees Celsius), with ad libitum access to food and water. The following transgenic mice were used: Thy1-ChR2-YFP (B6.Cg-Tg(Thy1-COP4/EYFP)18Gfng/J, Jackson Laboratory stock number: 007612), Gabra6-cre (B6.D2-Tg(Gabra6-cre)B1Lfr/Mmucd, MMRRC stock number: 015966-UCD), Gabra6-Cre (B6.129P2-Gabra6tm2(cre)Wwis/Mmucd, MMRRC stock number: 015968-UCD), R26-LSL-Gi-DREADD (B6.129-Gt(ROSA)26Sortm1(CAG-CHRM4*,-mCitrine)Ute/J, Jackson Laboratory stock number: 026219) and Ai32 (B6.Cg-Gt(ROSA)26Sortm32(CAG-COP4*H134R/EYFP)Hze/J, Jackson Laboratory (stock number: 024109).

Wild animals	This study did not involve wild animals.
Reporting on sex	We have used both male and female mice for almost all experiments in this study, since the neuronal mechanisms underlying delay eyeblink conditioning are expected to be highly similar between sexes. Only the in vivo whole-cell recordings have been performed in males for ethical reasons. Mice were individually housed after the first surgery and individual housing leads to extra discomfort in females, which was our motivation to use primarily males for this experiment. However, cerebellar nuclei neurons in female mice showed comparable Vm responses to male mice. Similarly, for all other main results of this paper, no differences were found between male and female mice, so we have pooled the data.
Field-collected samples	This study did not involve field-collected samples.
Ethics oversight	All animal experimental procedures were conducted in accordance with the institutional animal welfare committees of the Erasmus Medical Center, the Royal Dutch Academy of Arts and Sciences, the Champalimaud Centre for the Unknown or the Direcçãoo Geral de Veterinária (Ref. No. 0421/000/000/2015). All experiments adhered to the European guidelines for the care and use of laboratory animals (Council Directive 86/6009/EEC).

Note that full information on the approval of the study protocol must also be provided in the manuscript.
